# Polybrominated Diphenyl Ethers in Maternal Serum, Breast Milk, Umbilical Cord Serum, and House Dust in a South Korean Birth Panel of Mother-Neonate Pairs

**DOI:** 10.3390/ijerph13080767

**Published:** 2016-07-28

**Authors:** Mi-Yeon Shin, Sunggyu Lee, Hai-Joong Kim, Jeong Jae Lee, Gyuyeon Choi, Sooran Choi, Sungjoo Kim, Su Young Kim, Jeongim Park, Hyo-Bang Moon, Kyungho Choi, Sungkyoon Kim

**Affiliations:** 1 Graduate School of Public Health, Seoul National University, Seoul 151-742, Korea; damage7@snu.ac.kr (M.-Y.S.); kyungho@snu.ac.kr (K.C.); 2 Department of Marine Sciences and Convergent Technology, Hanyang University, Ansan 426-791, Korea; mealsgl@gmail.com (S.L.); hbmoon@hanyang.ac.kr (H.-B.M.); 3 College of Medicine, Korea University, Seoul 136701, Korea; haijkim@gmail.com; 4 College of Medicine, Soonchunhyang University, Seoul 140-743, Korea; jjlee@schmc.ac.kr (J.J.L.); kychoi@schmc.ac.kr (G.C.); 5 College of Medicine, Inha University, Incheon 402-751, Korea; csran@inha.ac.kr; 6 College of Medicine, Hallym University, Anyang 431-796, Korea; icastle@hallym.ac.kr; 7 College of Medicine, Jeju National University, Jeju 690-756, Korea; suy0202@jejunu.ac.kr; 8 College of Natural Sciences, Soonchunhyang University, Asan 336-745, Korea; jeongim@sch.ac.kr; 9 Institute of Health and Environment, Graduate School of Public Health, Seoul National University, 1 Gwanak-ro, Gwanak-gu, Seoul 151-742, Korea

**Keywords:** polybrominated diphenyl ethers, house dust, maternal serum, umbilical cord serum, breast milk, pregnant women

## Abstract

Polybrominated diphenyl ethers (PBDEs) have been used as flame retardants. Although many reports have indicated an association between exposure to PBDEs and developmental neurotoxicity, the relative contributions of different sources of dust PBDE congeners to the levels in various tissues of mother–baby pairs is not well understood. The aims of this study were thus to measure the quantitative relationship between the level of PBDEs in house dust and tissues of mother-neonate pairs, and to investigate the chemical sources of the PBDEs. Forty-one mother-neonate pairs were recruited and provided samples of maternal serum (*n* = 29), umbilical cord serum (*n* = 25), breast milk (*n* = 50), and house dust (*n* = 41), where PBDEs were determined with high-resolution gas chromatography coupled with high-resolution mass spectrometry. While deca- (e.g., BDE 209, detected 100%), nona- (BDE 206/207, 95.1%), octa- (BDE 183, 100%), penta- (BDE 99/153, 100%, 98%) and tetra-BDEs (BDE 47, 100%) were detected abundantly in dust, penta- (BDE 99, 76%, 92%) and tetra-BDEs (BDE 47, 84%, 98%) were detected abundantly in umbilical cord serum and breast milk, respectively; tetra-BDEs (BDE 47, 86%) were detected more often relative to other congeners in maternal serum. Spearman’s pairwise comparison showed that the levels of BDE 47 (ρ = 0.52, *p* < 0.001) and −99 (ρ = 0.64, *p* < 0.01) in umbilical cord serum were associated with BDE 209 levels in dust; BDE 47 in maternal serum also showed correlation with BDE 99 in cord serum (ρ = 0.48, *p* < 0.01) but there was no significant correlation between maternal BDE 47 and dust BDE 209. On the other hand, a comparison of the distribution among congeners suggested probable associations of BDE 47 in maternal serum, breast milk, and umbilical cord serum with BDE 209 in dust; and of BDE 99 in maternal and umbilical cord serum, breast milk, and dust with BDE 209 in dust. Although further studies are needed, a radar chart-based distributional comparison among congeners supported associations between BDE 47 or −99 in human tissues and BDE 209 in dust.

## 1. Introduction

Polybrominated diphenyl ethers (PBDEs), a group of synthetic organic chemicals with 209 congeners, have been widely used as chemical flame retardants for several decades. Three commercial PBDE formulations have been produced: pentabromodiphenyl ethers (penta-BDEs), octabromodiphenyl ethers (octa-BDEs) and decabromodiphenyl ethers (deca-BDEs), but deca-BDEs was reported to comprise 82% of the PBDE present in electronic products, electrical appliances and automotive industry products globally [1]. While octa-BDEs has been added to plastics for electronic equipment, in recent decades penta-BDEs has been used in cushions and mattresses along with polyurethane [2]. Owing to the stable chemical structure and strong tendency for bio-accumulation of PBDEs, they have been found in many biota specimens as persistent pollutants, and are regarded as endocrine disruptors [3,4,5,6]. Animal studies have suggested that PBDEs can affect liver function [7] and neurodevelopment [8], disrupt the endocrine system [9], and alter hormone levels [10]; similar health effects were also found in some epidemiological studies [11,12,13,14]. Recently, a report showed a positive association between PBDEs in human breast milk and congenital cryptorchidism in newborn boys [15]. Also, significant associations of prenatal exposure to PBDEs with poorer attention and executive function were investigated [16]. Owing to growing concerns about PBDEs exposure, penta- and octa-BDEs containing products have been banned or voluntarily phased out in Europe and the U.S.; however, the use of deca-BDEs and the recycling of PBDE products are still allowed in those countries including South Korea [17]. 

Regarding sources of exposure to PBDEs, physiological burden appears to be associated with their presence in food [18,19] and dust [20,21,22]. Considering that the indoor levels of PBDEs are usually higher than outdoor levels [23,24], house dust has been suggested as the main source of exposure to PBDEs [25,26,27,28]. According to a recent study, the estimated daily intake ratio between seafood and house dust differs by country: in South Korea and Belgium, this ratio is approximately 50:50, while in China and the US, house dust ingestion was found to be fourfold higher than seafood consumption in adults [17]. Such regional differences might be associated with different contributions from various sources. In South Korea, it has been asserted that seafood consumption and dust ingestion contribute equally to the total PBDEs intake in adults [17], while dust ingestion was proposed to be the major contributor in toddlers [18].

In the present study, we measured PBDEs in matched blood, breast milk, and dust samples from mother–neonate pairs to assess the relative levels of PBDEs in these media. To the best of our knowledge, this is the first study on the relationship between house dust and the physiological burden conferred by PBDEs in the context of fetal and maternal exposure in South Korea.

## 2. Materials and Methods

### 2.1. Study Population and Sample Collection

Forty-one mother-neonate pairs were recruited before delivery from five university hospitals, located in Ansan, Jeju, Pyungchon, and Seoul in South Korea, from February 2011 to December 2011. We only included healthy subjects without histories of thyroid disease. We collected maternal (*n* = 29) and umbilical cord blood (*n* = 25) from 29 mothers, resulting in 25 paired blood samples. Blood samples were collected in heparinized tubes during delivery. Blood from each individual was separated into aliquots on site and stored in polypropylene cryotubes at −70 °C until analysis. Breast milk samples were collected from 18 lactating women at 7, 15, and 30 days postpartum, in polypropylene tubes following a detailed standard operating protocol with pictorial guide. Before the mothers collect their breast milk, they were asked to wash the hands and wipe their breast with an alcohol cotton swab. Only 16 of these women had their breast milk collected at all three collection times; the remaining two had it collected only once. Therefore, a total of 50 breast milk samples were collected.

We visited the participants’ homes to collect domestic vacuum cleaner bags (*n* = 41) at June 2011 to July 2011. Participants we asked two weeks before the home visit to clean their living rooms and bedrooms everyday using their vacuum cleaner. After the vacuum cleaner bags were transported to the laboratory these bags were opened carefully. Hair and non-dust particles were removed, sieved with a mesh micron ≥100 µm, and kept at −20 °C until analysis. Personal information pertaining to lifestyle, diet, and demographics was obtained by a face-to-face interview and questionnaires during the home visits. Information on prenatal care and health status was extracted from medical case report forms. The protocol used in this study was approved by the Institutional Review Board (IRB) of the School of Public Health, Seoul National University (IRB No. 131-2011-02-14). All participants signed informed consent forms before participating.

### 2.2. Chemical Analysis

The experimental procedures for the analysis of PBDEs in human samples were optimized by making some modifications to those used in previous studies [29,30]. In brief, after ^13^C-labeled PBDEs were spiked, 2-mL blood serum or breast milk samples were fortified with formic acid and Milli-Q water for protein denaturation. The samples were extracted by solid-phase extraction (SPE) using Sep-Pak C_18_ SPE cartridges, which were pre-washed with MeOH and conditioned with Milli-Q water. The extracted cartridges were rinsed with Milli-Q water and subsequently dried. A Sep-Pak Plus NH_2_ cartridge, pre-washed with 6 mL of hexane, was connected to the lower end of the C_18_ cartridge. Hexane (8 mL) was passed through the combined NH_2_-C_18_ cartridges and collected. After removing the C_18_ cartridge, 6 mL of 5% dichloromethane (DCM) in hexane was passed through the NH_2_ cartridge and combined with the previous fraction. The pooled eluents were cleaned by a silica gel/Florisil SPE cartridge, using 12 mL of 50% DCM in hexane. The purified eluents were then concentrated and dissolved in 100 µL of nonane for instrumental analysis. PBDE concentrations were normalized by the lipid weight of serum or breast milk. Triglyceride and total cholesterol were determined by enzymatic methods in a commercial clinical laboratory, and the serum concentration of total lipids was calculated as follows: Total lipids (mg/dL) = (2.27 × total cholesterol (mg/dL)) + triglyceride (mg/dL) + 62.3 [31,32]. 

Levels of PBDEs in dust were determined using a previously published method [17]. Briefly, the dust samples (approximately 1 g) were extracted in a Soxhlet apparatus using 200 mL of 50% DCM (ultra-residue analysis; J. T. Baker, Phillipsburg, NJ, USA) and hexane (ultra-residue analysis; J. T. Baker) (1:1, *v*:*v*) for 20 h. Before the extraction, 2 ng of surrogate standards (MBDE-MXE; Wellington Laboratories, Guelph, ON, Canada) was spiked into the samples. The extracts were then concentrated to 1–2 mL using a rotary evaporator. Next, the dust sample extracts were cleaned by passage through a multi-layer silica gel column with 150 mL of 15% DCM in hexane using the Dioxin Cleanup System (DAC695/DPU8; GL Sciences, Tokyo, Japan). The eluents were concentrated to approximately 1 mL and then evaporated at room temperature to 50–100 µL. The residues were dissolved in 100 µL of nonane for instrumental analysis. Twenty-one PBDE congeners (BDE 17, −28, −47, −66, −71, −85, −99, −100, −119, −126, −153, −154, −183, −184, −190, −191, −196, −197, −206, −207 and −209) composed of tri- to deca-BDEs were measured.

### 2.3. Instrumental Analysis and Quality Control

High-resolution gas chromatography interfaced with a high-resolution mass spectrometer (HRGC/HRMS; JMS 800D; JEOL, Tokyo, Japan) was used for the identification and quantification of PBDEs. Details of the instrumental parameters are reported elsewhere [33,34]. In brief, PBDEs were quantified using the isotope dilution method based on relative response factors of individual compounds. The HRMS was operated under positive electron ionization mode, and ions were monitored by selected ion monitoring using molecular ions of target compounds. A DB5-MS (15 m length, 0.25 mm internal diameter, 0.1 µm film thickness; J & W Scientific, Palo Alto, CA, USA) column was used for the separation of individual PBDE congeners. The limit of quantitation (LOQ), calculated according to the mean serum lipid content of a 2-mL serum sample, ranged from 0.17 (BDE 17 to −126) to 0.83 (BDE 138 to −191) ng/g lipid weight. For dust samples, the LOQ was calculated as 10 times the signal-to-noise ratio, which ranged from 0.7 to 3.0 ng/g d.w. for tri- to nona-BDEs, and 30 ng/g d.w. for deca-BDEs. The recovery rates of ^13^C-labeled surrogate standards of PBDEs were 86% ± 19% for house dust and 87% ± 13% for human samples. To assess the quality of PBDE determination, standard reference house dust materials (SRM 2585; NIST, Gaithersburg, MD, USA) were analyzed. The levels of accuracy (*n* = 5) of the measured values for tri- to hepta-BDEs and octa- to deca-BDEs were 90% ± 10% (mean ± standard deviation (SD)) and 82% ± 12%, respectively. A mid-point calibration standard was injected to check for instrumental drift in sensitivity after every 15 samples. The results showed a coefficient variation of <10% for all congeners of PBDEs. Solvents injected before and after the injection of standards showed negligible contamination or carryover. Procedural blanks (*n* = 10) were processed with each set of 15 serum samples to check for laboratory contamination. Blanks did not contain quantifiable amounts of target contaminants. 

### 2.4. Statistical Analysis

Levels of PBDEs in biological samples and dust were natural-log-transformed before statistical analysis due to their log-normal distribution; the levels of PBDEs below the LOQ were assigned a value of the LOQ divided by the square root of 2. For calculation of the total PBDE level (ΣPBDEs; sum of the PBDE congeners determined for each subject), we replaced the values below the LOQ with zero, to prevent over-inflation caused by summation of inputted congener levels that were below the LOQ [20]. To evaluate the association between the levels of PBDE congeners and ΣPBDEs of the different media, nonparametric correlation statistics (Spearman’s rank) were used. To analyze the difference between the groups, ANOVA was used. To minimize the effects of outliers, including on the regression models, candidate outliers were assessed with the PROC UNIVARIATE function in SAS using a trim option of 0.1. In the model, the maximum level of BDE 47 in maternal serum and house dust was trimmed before regression modeling [35]; scatterplots were also produced including trimmed data. (Figure S1). Regarding the assessment of the association among congeners, we used a simple correlation analysis as well as a comparison of the distribution of each congener. While Spearman’s correlation was useful to measure the relationship among congeners matched in sample media, radar chart-like graphics allowed us to compare the overall association among congeners including unmatched cases. All data were natural-log transformed due to skewness. Then, each congener by media was standardized to have a mean of zero and standard deviation of unity so as to make relative comparison among congeners in different media. Because the levels of BDEs were widely distributed by congener and medium, and varied by orders of magnitude, standardization of the measurements was necessary before the levels of the different congeners could be compared. In the radar chart, one was used as a reference and the others were used as a comparison group. Since our assumption was that less brominated congeners in dust or biological samples were derived from more highly brominated congeners, most references were highly brominated congeners in the plots. If a reference congener had a high correlation with the comparison congeners, their distributions should overlap. Subsequently, we sorted the data array by the reference congener and obtained spiral plots showing the reference congeners in ascending order. In the case of a strong association between the reference and comparison congeners, the comparison congeners followed the spiral lines more exactly. To avoid overly complicated radar charts, congeners were grouped based on their covariance estimates (“between-congener variance components”), which were derived in preliminary mixed-effect models (where the dependent variables were standardized PBDE levels with a random intercept of congener by sample medium, especially for PBDEs frequently detected in biological media as random blocks (indicating small mixed effects; e.g., BDE 47, −99, −153 and −183). There was no statistically significant difference in the congener levels of breast milk collected at the three different time points in the preliminary analysis. While summary statistics used individual measurements, covariance estimation and radar charts used subject-specific means. All statistical analyses were performed using SAS software (ver. 9.3, SAS Institute, Cary, NC, USA). 

## 3. Results

### 3.1. PBDEs in House Dust

**Figure 1:**
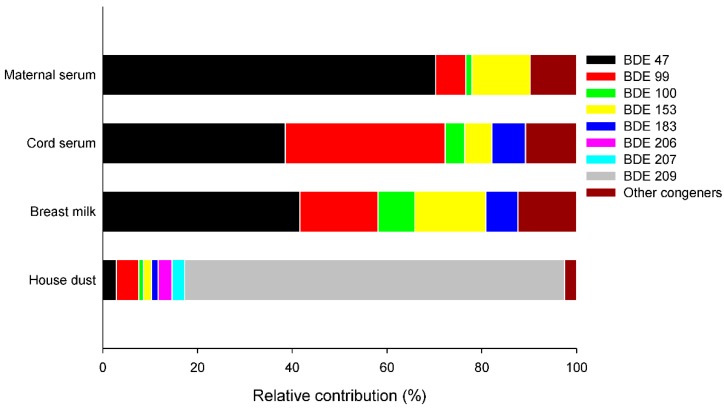
Relative amounts of PBDE congeners in human samples and house dust.

**Figure 2:**
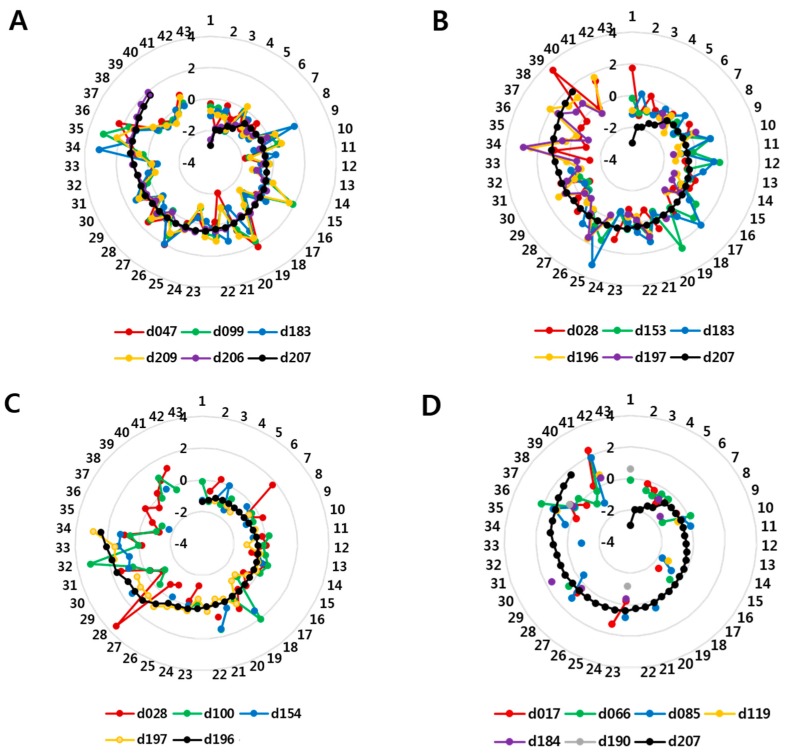
Radar charts of major polybrominated diphenyl ether (PBDE) congeners detected in house dust. (**A**) deca-BDEs, nona-BDEs, and less brominated BDEs in dust showing the strongest associations (${\hat{\sigma }}_{b.dust}^{2}$: 0.457–0.459); (**B**) nona-BDEs and less brominated BDEs in dust showing strong associations (${\hat{\sigma }}_{b.dust}^{2}$: 0.438–0.459); (**C**) octa-BDEs (BDE 196) and less brominated BDEs (${\hat{\sigma }}_{b.dust}^{2}$: 0.438–0.454) in dust showing weak associations; (**D**) nona-BDEs and less brominated BDEs (${\hat{\sigma }}_{b.dust}^{2}$: 0.150–0.416) in dust showing the weakest associations. The values from the center indicate log transformed PBDE levels, standardized with a mean of zero and standard deviation of unity, and those around the circumference are observation numbers in ascending order of reference BDE (207 or −196) levels, where the congener number is provided in the legend after the “d” character. For example “d047” corresponds to BDE 47 in dust.

The most abundant congener was BDE 209, which constituted 80.2% of the ΣPBDEs detected. This was followed by BDE 99 (4.7%), −47 (2.9%), −206 (2.9%) and −207 (2.7%) (Table 1, Figure 1). Some of them within and between commercial groups showed significantly high correlations ranging from *p* < 0.0001–0.0009: *ρ* = 0.85–0.96 for deca- to nona-BDEs (BDE 206, −207); *ρ* = 0.51 for nona- (BDE 207) to tetra-BDEs (BDE 47); *ρ* = 0.67–0.77 for nona/deca- to octa-BDEs (BDE 196); *ρ* = 0.53 for nona- (BDE 206) to octa-BDEs (BDE 183); *ρ* = 0.56–0.61 for octa- (BDE 183) to penta-BDEs (BDE 99, −100, −153); *ρ* = 0.54 for octa- (BDE 183) to tetra-BDEs (BDE 47); and *ρ* = 0.67–0.93 for penta- (BDE 85, −99, −100, −153, −154) to tetra-BDEs (BDE 47, −66) (Table S1), and they were depicted consistently with radar charts (Figure 2 and Figure S2).

### 3.2. PBDE Levels in Biological Samples

Although PBDEs were detected in most biological media based on the ΣPBDEs data, the highest geometric mean amount within the first week after birth was in umbilical cord serum (7.37 ng/g lipid; 95% confidence level: 1.07–2.20), followed by maternal serum (1.93 ng/g lipid; 95% CL: 1.43–2.59) and breast milk (7 days postpartum; 1.53 ng/g lipid (95% CL: 1.07–2.19), 15 days postpartum; 1.64 ng/g lipid (95% CL: 1.15–2.35), 30 days postpartum, 1.50 ng/g lipid (95% CL: 0.98–2.28) (Table 1). The levels of individual PBDE congeners and the ΣPBDEs did not differ significantly according to the breast milk collection time (*p* = 0.924). 

While BDE 47 was most abundant in maternal serum, followed by BDE 153, the congener composition was notably different depending on the biological matrix (Figure 1). Increased levels and detection rates of BDE 99 were observed in umbilical cord serum, and the ratio between BDE 47 and −99 in this matrix was approximately 1:1. In breast milk, most abundant congener was BDE 47 followed by −99 and −153.

### 3.3. Association among PBDE Congeners in Human Samples and House Dust

**Figure 3:**
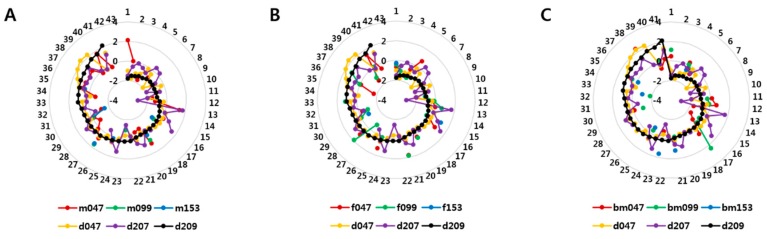
Radar charts of major PBDE congeners detected in house dust and human tissues. (**A**) Levels of brominated diphenyl ethers (BDEs) in maternal serum and levels of BDE 209, −207 and −47 in dust; (**B**) Levels of BDEs in umbilical cord serum and levels of BDE 209, −207 and −47 in dust; (**C**) Levels of BDEs in breast milk and levels of BDE 209, −207 and −47 in dust. The values from the center indicate log transformed PBDE levels, standardized with a mean of zero and standard deviation of unity, and those around the circumference are observation numbers in ascending order of reference BDE (d209) levels, where the congener number is provided in the legend after “m, f, bm and d” character. m; maternal serum, f; umbilical cord serum, bm; breast milk, d; house dust. For example “m047” corresponds to BDE 47 in maternal serum.

There were considerable associations between BDE 47 and −99 in umbilical cord serum and BDE 209 levels in house dust. Based on pairwise comparison, significant correlations were found between BDE 209 in dust and BDE 47 (*p* < 0.001) and −99 (*p* < 0.01) in umbilical cord serum (Table S2, Figure S1) although BDE 47 levels in maternal serum did not show this trend (*p* > 0.1, Figure S1). To the contrary, the relative distributions in radar charts depicted tight spiral lines of highly brominated BDEs in dust some congeners in cord serum. The spiral lines of BDE 47, −99 in cord serum were regressed upon the corresponding BDE 209 in dust (Figure 3) and BDE 207. Comparatively, lines for BDE 47 and −99 in breast milk and BDE 47 in maternal serum followed the reference BDEs in dust (e.g., BDE 47, −99 and 153; Figure 3, Figure S3).

## 4. Discussion

Diet and house dust have been considered as the main sources of BDEs in human tissues [24,36]; the most abundant congeners in human tissues have been reported as BDE 47 and other less brominated BDEs, while the most abundant congeners in house dust were BDE 209 and other more highly brominated BDEs [21,37,38,39]. In the present study, we measured PBDE congener levels in different media of matched mother-neonate pairs to assess the contribution of house dust PBDEs to those levels.

### 4.1. PBDEs in House Dust

**Figure 4:**
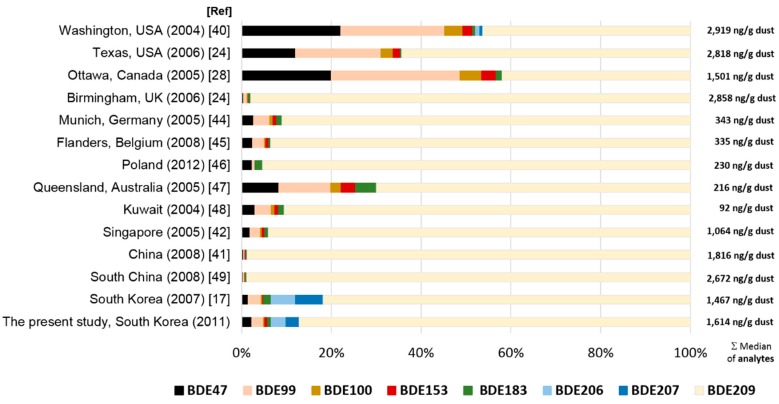
Relative amounts of PBDE congeners in house dust (% of median dry weight (ng/g)) among different countries [17,24,28,40,41,42,44,45,46,47,48,49].

Most congeners were detected in dust, but BDE 209 was by far the most abundant (80.2% of ΣPBDEs), followed by BDE 206/207 (5.6%), −99 (4.7% of ΣPBDE) and −47 (2.9% of ΣPBDE). In the present study, ΣPBDEs in house dust, and the proportions of individual congeners, agreed with a previous study conducted in South Korea [17]. Among several different countries, ΣPBDEs was highest in the U.S. [40] (Figure 4), which showed a similar ΣPBDE to that of the UK [24]. The ΣPBDEs in the present study was lower than in the UK and the U.S., but higher than in Oceania [24,39]. The ΣPBDEs in this study was most similar to those in other East Asian countries [41,42]. Although the use of penta-BDEs and octa-BDEs in consumer products has been restricted internationally since 2004 (they have since started to be replaced by other flame retardants), both penta- and octa-BDEs have been detected at high concentrations in house dust [43].

In dust samples, the levels of congeners in each homologous group were significantly correlated (deca-BDEs: Spearman’s *r* = 0.85 for tetra-; *r* = 0.69–0.87 for penta-; *r* = 0.67–0.83 for hexa-; *r* = 0.54 for hepta-; and *r* = 0.51 for nona-BDEs; Table S1), as illustrated in the radar charts (Figure 2, Figure S2). Our data were standardized with the same mean (zero) and standard deviation (one); therefore, the close variance components would indicate a strong association among congener groups. The radar chart shows that BDE 183 and −153 followed the spiral lines of BDE 207 and −209 exactly (Figure 2A,B), whereas BDE 28, −100 and −154 followed the spiral lines of BDE 207, −196 and −197 less exactly (Figure 2B,C; ${\hat{\sigma }}_{b.dust}^{2}$, 0.438–0.454); however, there seemed to be no association between BDE 85, −66, and −17 (${\hat{\sigma }}_{b.dust}^{2}$, 0.411–0.454) and BDE 207 (Figure 2D).

Although this was not a mechanistic experiment, a strong agreement among congeners in house dust suggest that less brominated congeners such as BDE 47, −99 and −183 in dust are produced from highly brominated congeners (BDE 209, −207 and −206) as degradation products (Figure 2, Figure S2). In addition to strong associations among the levels of certain congeners, some congeners were associated with a higher proportion of other, more highly brominated compounds, which was probably attributable to the degradation of these compounds into less brominated congeners, or to direct input from other sources; however, random fluctuation or swing movement across mainly BDE 209, −207 and −206, as well as −183 or −153, might indicate that disposition (or breakdown, i.e., debromination) of highly brominated congeners is the main factor contributing to the smaller congeners. The debromination of BDE 209 into less brominated congeners in house dust has been shown previously [23,50].

### 4.2. PBDE Levels in Biological Samples of Mother-Neonate Pairs

BDE 47 was the most abundant congener detected in all bio-samples; this agrees with previous studies [38,51,52]. Its abundance could be explained by relatively long half-life (1.8 years) in humans [53] and possibly frequent exposure through consumption of foods which also have abundant BDE 47 [54,55]. Our results regarding blood BDE 47 levels were comparable to those of other South Korean studies of mother-neonate pairs. The previous studies, which had smaller sample sizes, reported BDE 47 levels of 0.97–60.0 ng/g lipid for maternal serum, 2.06–230 for umbilical cord serum, and 0.37–180 for breast milk [56,57]. The median concentrations of BDE 47 in serum in our study were also similar to those determined in previous European and Asian studies [25,58,59,60], but lower than those in U.S. studies [51,61]. The levels of individual congeners, and the ΣPBDEs, in umbilical cord serum were significantly higher than the levels in maternal serum, which is consistent with several previous reports [58,62,63]. This could be explained by the lower capacity for chemical metabolism by the fetus, resulting in greater concentrations of highly brominated PBDEs in umbilical cord serum than in maternal serum [62]. There was a significant (*p* < 0.01) association between maternal serum for BDE 47 and umbilical cord serum for BDE 99; although the levels of other congeners in the two media were also likely to be related, the low detection rates (due to small sample volumes) were likely responsible for no further relationships being identified. This finding is supported by previous evidence of significant correlations between maternal and fetal matrices in terms of BDE 47 levels and ΣPBDEs [51,60,64], which strongly suggests that the placenta is not an effective barrier to limit the transport of PBDEs. Meanwhile, the levels of BDE 47, 99 and −100 in breast milk were not significantly correlated with fetal nor maternal serum.

### 4.3. Correlations between Human Samples and House Dust

We collected a house dust sample from each participant at different time points. In total, 60% of participants provided house dust samples during the prenatal period; the remaining participants provided a postnatal sample. Variability in the levels of PBDEs in dust, in the same house according to sampling time (with measurements separated by an interval of approximately 5 years) and season, were previously reported [65]; however, low variability was observed between samples separated by less than 1 year [23]. Because the interval between sampling times was very short (June 2011 to July 2011) in the present study, we considered that the PBDE levels in house dust would not have been affected by sampling time or season.

Although contaminated food and dust have been considered as the main sources of PBDEs in human tissues, we found significant correlations between BDE 47 and −99 in umbilical cord serum and BDE 209 levels in dust; however, BDE 47 in maternal serum and BDE 47, −99, −100 in breast milk were not correlated with dust BDE 209, which could be due to data sparseness. Pairwise comparison showed the correlation between matched analytes only; missing samples or incomplete dataset wastes the unmatched observations. In order to overcome this limitation, we devised radar charts with all observations to show order-sorted distribution relative to ones of other analytes. These radar charts suggested association among variables with both matched and unmatched observations. In the case of strong association among variables, all observations of comparison variables followed the reference variable (BDE 209 in dust) tightly as shown in Figure 2A. The limited number of observations in our pairwise comparison analyses probably obscured the true association; however, the radar charts suggested considerable associations among abundant congeners (BDE 47, −99 and −153) in maternal or fetal tissue and deca-BDEs (or nona-BDEs) and those fetal tissue (Figure 3, Figure S3). Interestingly, there was a strong association between BDE 47 and deca- or nona-BDEs levels in dust, which suggests that BDE 209 can be broken down into BDE 47 in dust. In contrast, the spiral lines of high brominated congeners such as BDE 207 and −209 in house dust was close to the spiral lines of lower brominated congeners such as BDE 47, −99 and −153 in biological samples at low levels. However, those spiral lines of both house dust and biological samples drifted apart at high levels (Figure 3). Therefore, we speculated that high concentration in biological samples might be result from other sources not only house dust.

In vivo BDE 209 has a very short half-life (several days) compared with those of other congeners; thus, it may be more readily transformed into less brominated congeners, or eliminated [66] and eventually prevent reliable measurement in biological media. Stapleton, et al. [67] reported that, in carp fed BDE 209 alone, only less brominated congeners were present. Thuresson, et al. [68] detected less brominated congeners in workers who had been exposed mainly to deca-BDEs.

Because concentration of BDE 209 in house dust are high, human can be exposed continuously. We speculated that house dust could be an important source of PBDEs and may contribute to physiological burden of less brominated congeners found in humans.

This is the first study to assess the association between PBDE levels in matched maternal and fetal samples and house dust. Despite the limited sample size, the present study suggested considerably strong association between highly-brominated BDEs in dust and lower-brominated ones in biological samples although further researches are need to confirm.

## 5. Conclusions 

The results in the present study indicated that BDE 209, −206/207, −183, −99/153 and −47 were detected in most dust samples although BDE 99 and BDE 47 were detected abundantly in umbilical cord serum and breast milk, respectively; BDE 47 were detected more often relative to other congeners in maternal serum. According to pairwise comparison, there were significant correlation between BDE 47 and −99 in cord serum and BDE 209 in dust; BDE 47 in maternal serum and BDE 99 in cord serum but no significant correlation between maternal BDE 47 and dust BDE 209. However, distribution comparison with radar charts suggested probable associations of BDE 47 in maternal serum, breast milk, and umbilical cord serum with BDE 209 in dust; those of BDE 99 in maternal and umbilical cord serum, breast milk with BDE 209 in dust. Although further studies on toxicokinetics of dust BDE 209 in human tissues are needed, similar distribution and associations between BDE 47 or −99 in human tissues and BDE 209 in dust suggested considerable dosage of lower-brominated BDE in mother-neonate pairs might came from their house dust. 

## Figures and Tables

**Table 1:** Summary of PBDE concentrations in matched samples of house dust, maternal serum, umbilical cord serum, and breast milk in mother-newborn pairs.

	House Dust (*n* = 41)					Maternal Serum (*n* = 29)					Umbilical Cord Serum (*n* = 25)					Breast Milk ^a^ (*n* = 50)				
	(ng/g Dry Weight)					(ng/g Lipid Weight)														
Congener	min	50th	75th	max	%detect ^b^	min	50th	75th	max	%detect	min	50th	75th	max	%detect	min	50th	75th	max	%detect
tri-BDEs	<LOQ	15.5	22.6	239.3	80.5	<LOQ	<LOQ	<LOQ	0.1	3.4	<LOQ				-	<LOQ	<LOQ	<LOQ	0.4	16.0
BDE 17	<LOQ	<LOQ	1.6	8.7	34.1	<LOQ				-	<LOQ				-	<LOQ				-
BDE 28	<LOQ	14.8	21.6	239.3	80.5	<LOQ	<LOQ	<LOQ	0.1	3.4	<LOQ				-	<LOQ	<LOQ	<LOQ	0.35	16.0
tetra-BDEs	6.7	39.6	63.1	418.7	100	<LOQ	0.8	1.6	8.9	86.2	<LOQ	0.8	3.6	6.5	84.0	0.3	0.6	0.8	2.1	96.7
BDE 47	6.7	37.0	63.1	307.9	100	<LOQ	1.2	2.2	6.5	86.2	<LOQ	2.1	3.6	6.5	84.0	<LOQ	0.6	0.7	2.1	98.0
BDE 49	<LOQ				-	<LOQ	<LOQ	<LOQ	0.5	6.9	<LOQ	<LOQ	<LOQ	2.9	16.0	<LOQ	<LOQ	<LOQ	0.6	6.0
BDE 66	<LOQ	<LOQ	2.7	13.4	36.6	<LOQ	<LOQ	<LOQ	0.6	13.8	<LOQ	<LOQ	<LOQ	2.0	12.0	<LOQ	<LOQ	<LOQ	0.6	4.0
BDE 71	<LOQ	<LOQ	<LOQ	97.4	4.9	<LOQ	<LOQ	<LOQ	2.4	13.8	<LOQ	<LOQ	<LOQ	0.9	8.0	<LOQ	<LOQ	<LOQ	0.1	6.0
BDE 77	<LOQ				-	<LOQ	<LOQ	<LOQ	0.8	10.3	<LOQ	<LOQ	<LOQ	0.9	20.0	<LOQ	<LOQ	<LOQ	0.1	2.0
penta-BDEs	12.4	69.8	160.4	1615.1	100	<LOQ	<LOQ	<LOQ	10.4	27.6	<LOQ	<LOQ	3.8	17.3	76.0	<LOQ	0.9	1.5	17.0	92.0
BDE 85	<LOQ	<LOQ	2.4	14.1	36.6	<LOQ				-	<LOQ					<LOQ				-
BDE 99	6.7	41.2	83.5	1571.0	100	<LOQ	<LOQ	<LOQ	2.5	20.7	<LOQ	2.2	3.1	8.8	76.0	<LOQ	0.3	0.5	13.7	88.0
BDE 100	<LOQ	6.0	9.7	250.4	70.7	<LOQ	<LOQ	<LOQ	0.7	13.8	<LOQ	<LOQ	0.7	2.9	28.0	<LOQ	0.1	0.3	3.3	56.0
BDE 119	<LOQ	<LOQ	<LOQ	80.3	12.2	<LOQ	<LOQ	<LOQ	0.4	6.9	<LOQ					<LOQ	<LOQ	<LOQ	0.1	6.0
BDE 126	<LOQ	<LOQ	<LOQ	8.1	4.9	<LOQ	<LOQ	<LOQ	0.3	3.4	<LOQ					<LOQ	<LOQ	<LOQ	0.1	6.0
BDE 138	<LOQ				-	<LOQ				-	<LOQ	<LOQ	<LOQ	6.0	4.0	<LOQ				-
BDE 153	<LOQ	8.5	31.0	253.4	97.6	<LOQ	<LOQ	<LOQ	6.4	24.1	<LOQ	<LOQ	<LOQ	11.9	12.0	<LOQ	<LOQ	0.7	1.7	42.0
BDE 154	<LOQ	1.4	5.2	19.2	53.7	<LOQ	<LOQ	<LOQ	1.0	6.9	<LOQ	<LOQ	<LOQ	5.8	12.0	<LOQ				-
octa-BDEs	3.0	28.2	50.8	461.3	100					-	<LOQ	<LOQ	<LOQ	38.3	8.0	<LOQ	<LOQ	<LOQ	1.2	24.0
BDE 183	3.0	13.8	27.8	335.0	100	<LOQ				-	<LOQ	<LOQ	<LOQ	38.3	8.0	<LOQ	<LOQ	<LOQ	1.2	24.0
BDE 184	<LOQ	<LOQ	<LOQ	22.9	17.1	<LOQ				-	<LOQ				-	<LOQ				-
BDE 190	<LOQ	<LOQ	<LOQ	18.8	7.3	<LOQ				-	<LOQ				-	<LOQ				-
BDE 191	<LOQ	<LOQ	<LOQ	120.1	4.9	<LOQ				-	<LOQ				-	<LOQ				-
BDE 196	<LOQ	6.7	10.2	51.1	82.9	<LOQ				-	<LOQ				-	<LOQ				-
BDE 197	<LOQ	6.2	11.7	75.2	68.3	<LOQ				-	<LOQ				-	<LOQ				-
nona-BDEs	<LOQ	105.5	181.2	597.9	95.1	n.a ^c^				-	n.a				-	n.a				-
BDE 206	<LOQ	53.3	88.0	307.2	95.1	n.a				-	n.a				-	n.a				-
BDE 207	<LOQ	46.6	81.9	290.7	95.1	n.a				-	n.a				-	n.a				-
deca-BDEs	498.0	1407.8	2273.8	5706.8	100	n.a				-	n.a				-	n.a				-
(BDE 209)																				
Total BDE ^d^	645.8	2011.3	3165.3	6360.1	100	<LOQ	1.9	4.3	13.6	92.9	<LOQ	7.4	15.2	49.0	92.0	0.3	1.6	2.3	19.8	100

BDE, brominated diphenyl ether; octa-BDEs, octabromodiphenyl ethers; deca-BDEs, decabromodiphenyl ethers; LOQ, limit of quantification. ^a^ Breast milk sample data (min. 1 to max. 3) were averaged for each individual. ^b^ proportion of observations above LOQ. ^c^ Not analyzed due to small sample. ^d^ Total BDEs (ΣPBDEs) were calculated by summing values for all of the congeners analyzed in each matrix.

## Supplementary Materials

The following are available online at www.mdpi.com/1660-4601/13/8/767/s1. Table S1: Spearman’ correlation coefficient among PBDE congeners in house dust; Table S2: Spearman’ correlation coefficient between predominant congeners (>75% detection rate) in umbilical cord serum, maternal serum and nona/deca-BDEs in house dust; Figure S1: Scatter plots with regression lines between BDE 47, 99 and 100 in tissue and BDE 209 in house dust. All data were natural-log transformed and then, were standardized to have a mean of zero and standard deviation of unity. “m” and “f” mean maternal serum and umbilical cord serum, respectively. (ln_m47; β = 0.168, *p* > 0.1, ln_f47; β = 0.714, *p* < 0.01, ln_f99; β = 0.814, *p* < 0.001 and ln_f100; β = 0.834, *p* < 0.01); Figure S2: Radar charts of PBDE congeners detected in house dust by homologous group of PBDE formulations <extended>. The values from the center indicate log transformed PBDE levels, standardized with a mean of zero and standard deviation of unity, and those around the circumference are observation numbers in ascending order of reference BDE (black dots and lines) levels, where the congener number is provided in the legend after the “d” character. For example “d047” corresponds to BDE 47 in dust; Figure S3: Radar charts of major PBDE congeners detected in house dust and major congener detected in human tissues <extended>. The values from the center indicate log transformed PBDE levels, standardized with a mean of zero and standard deviation of unity, and those around the circumference are observation numbers in ascending order of reference BDE (d209) levels, where the congener number is provided in the legend after the “m, f, bm and d” character. m; maternal serum, f; umbilical cord serum, bm; breast milk, d; house dust. For example “m047” corresponds to BDE 47 in maternal serum.
