# Supplementary material for: Polybrominated Diphenyl Ethers in Maternal Serum, Breast Milk, Umbilical Cord Serum, and House Dust in a South Korean Birth Panel of Mother-Neonate Pairs
Source: Int J Environ Res Public Health. 2016 Jul 28;13(8):767. doi: 10.3390/ijerph13080767 (PMC4997453; doi:10.3390/ijerph13080767)
Supplement: Supplementary file 1 [file ijerph-13-00767-s001.pdf]

Supplementary Materials: Polybrominated Diphenyl Ethers in Maternal Serum, Breast Milk, Umbilical Cord Serum, and House Dust in a South Korean Birth Panel of Mother–Neonate Pairs

Mi-Yeon Shin, Sunggyu Lee, Hai-Joong Kim, Jeong Jae Lee, Gyuyeon Choi, Sooran Choi, Sungjoo Kim, Su Young Kim, Jeongim Park, Hyo-Bang Moon, Kyungho Choi and Sungkyoon Kim

Table S1. Spearman' correlation coefficient among PBDE congeners in house dust.

BDE congeners		Tri-	Tetra-		Penta-				Hexa-		Hepta-		Octa-		Nona-		Deca-
		**28**	**47**	**66**	**85**	**99**	**100**	**119**	**153**	**154**	**183**	**184**	**196**	**197**	**206**	**207**	**209**
**17**	Spearman's (ρ)	0.692	0.591	0.572	0.428	0.402	0.636	−0.500	0.446	0.600	0.573	1.000	0.636	0.633	0.412	0.405	0.393
	p-value	0.006	0.026	0.065	0.337	0.154	0.026	0.666	0.109	0.087	0.032	<0.0001	0.035	0.067	0.182	0.190	0.164
	n	14	14	11	7	14	12	3	14	9	14	5	11	9	12	12	14
**28**	Spearman's (ρ)		0.245	−0.064	0.358	0.046	0.491	−0.100	0.048	0.269	−0.009	0.142	0.085	0.121	0.127	0.094	0.303
	p-value		0.167	0.819	0.208	0.798	0.010	0.872	0.792	0.278	0.957	0.787	0.676	0.590	0.495	0.612	0.085
	n		33	15	14	33	26	5	32	18	33	6	26	22	31	31	33
**47**	Spearman's (ρ)			0.846	0.871	0.827	0.679	0.900	0.674	0.832	0.539	0.821	0.475	0.390	0.478	0.510	0.424
	p-value			<0.0001	<0.0001	<0.0001	<0.0001	0.037	<0.0001	<0.0001	0.0003	0.023	0.004	0.040	0.002	0.0009	0.005
	n			15	15	41	29	5	40	22	41	7	34	28	39	39	41
**66**	Spearman's (ρ)				0.821	0.753	0.829	1.000	0.657	0.927	0.700	1.000	0.461	0.103	0.379	0.318	0.478
	p-value				0.023	0.001	0.0005	<0.0001	0.007	<0.0001	0.003	<0.0001	0.112	0.777	0.201	0.288	0.071
	n				7	15	13	3	15	11	15	3	13	10	13	13	15
**85**	Spearman's (ρ)					0.500	0.637	0.900	0.578	0.853	0.221	0.900	0.520	0.300	0.494	0.478	0.571
	p-value					0.057	0.019	0.037	0.023	0.0004	0.427	0.037	0.056	0.342	0.085	0.098	0.026
	n					15	13	5	15	12	15	5	14	12	13	13	15
**99**	Spearman's (ρ)						0.548	−0.100	0.554	0.795	0.561	0.750	0.396	0.257	0.456	0.451	0.368
	p-value						0.002	0.872	0.0002	<0.0001	0.0001	0.052	0.020	0.185	0.003	0.004	0.017
	n						29	5	40	22	41	7	34	28	39	39	41
**100**	Spearman's (ρ)							0.900	0.582	0.525	0.602	0.028	0.465	0.215	0.486	0.461	0.376
	p-value							0.037	0.0009	0.025	0.0005	0.957	0.022	0.374	0.010	0.015	0.043
	n							5	29	18	29	6	24	19	27	27	29
**119**	Spearman's (ρ)								0.200	0.400	0.300	1.000	0.300	0.200	0.800	0.800	0.900
	p-value								0.747	0.600	0.623	.	0.623	0.800	0.200	0.200	0.037
	n								5	4	5	2	5	4	4	4	5
**153**	Spearman's (ρ)									0.559	0.606	−0.142	0.359	0.511	0.342	0.333	0.205

	p-value	0.006	<0.0001	0.759	0.036	0.005	0.035	0.040	0.203
	n	22	40	7	34	28	38	38	40
**154**	Spearman's (ρ)		0.601	0.714	0.756	0.535	0.754	0.729	0.681
	p-value		0.003	0.110	0.0001	0.039	0.0001	0.0003	0.0005
	n		22	6	20	15	20	20	22
**183**	Spearman's (ρ)			0.892	0.589	0.518	0.531	0.496	0.392
	p-value			0.006	0.0002	0.004	0.0005	0.001	0.011
	n			7	34	28	39	39	41
**184**	Spearman's (ρ)				0.750	0.700	0.714	0.771	0.785
	p-value				0.052	0.188	0.110	0.072	0.036
	n				7	5	6	6	7
**196**	Spearman's (ρ)					0.902	0.765	0.692	0.670
	p-value					<0.0001	<0.0001	<0.0001	<0.0001
	n					28	32	32	34
**197**	Spearman's (ρ)						0.563	0.528	0.433
	p-value						0.002	0.004	0.021
	n						27	27	28
**206**	Spearman's (ρ)							0.959	0.870
	p-value							<0.0001	<0.0001
	n							39	39
**207**	Spearman's (ρ)								0.849
	p-value								<.0001
	n								39

Table S2. Spearman' correlation coefficient between predominant congeners (>75% detection rate) in umbilical cord serum, maternal serum and nona/deca-BDEs in house dust.

			**House Dust**		
**Human tissues**	**BDE congener**		**206**	**207**	**209**
**Maternal serum**	**47**	Spearman's (ρ)	0.241	0.243	0.242
		p-value	0.257	0.253	0.245
		n	24	24	25
**Umbilical cord serum**	**47**	Spearman's (ρ)	**0.447**	**0.502**	**0.523**
		p-value	0.007	0.005	<0.001
		n	18	18	19
	**99**	Spearman's (ρ)	**0.573**	**0.604**	**0.637**
		p-value	0.013	0.008	0.003
		n	18	18	19

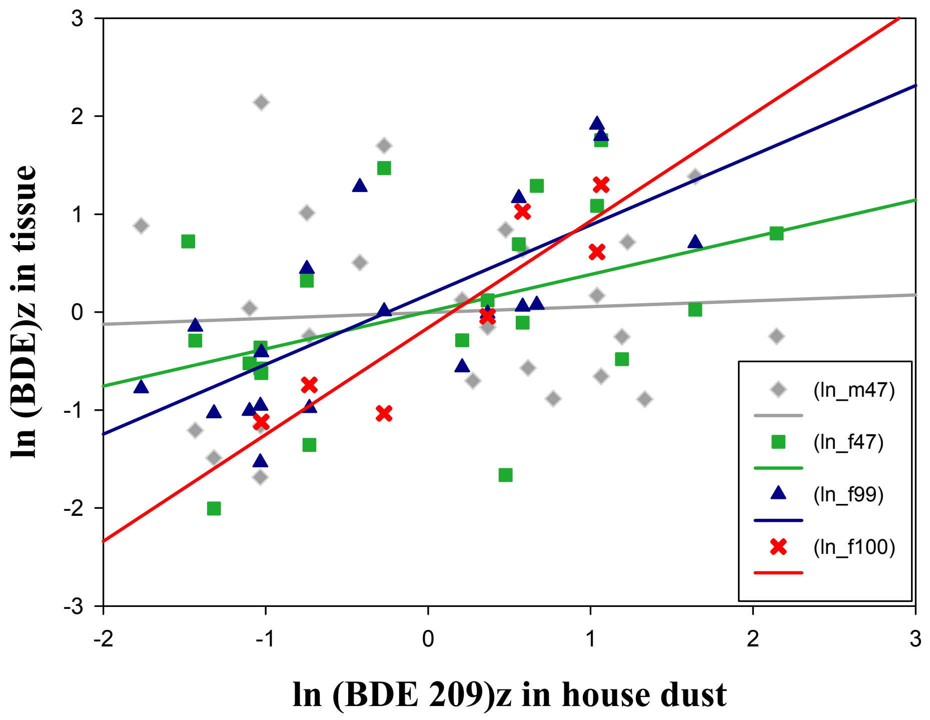

Figure S1. Scatter plots with regression lines between BDE 47, 99 and 100 in tissue and BDE 209 in house dust. All data were natural-log transformed and then, were standardized to have a mean of zero and standard deviation of unity. "m" and "f" mean maternal serum and umbilical cord serum, respectively. (ln_m47; $\beta = 0.168$, $p > 0.1$, ln_f47; $\beta = 0.714$, $p < 0.01$, ln_f99; $\beta = 0.814$, $p < 0.001$ and ln_f100; $\beta = 0.834$, $p < 0.01$).

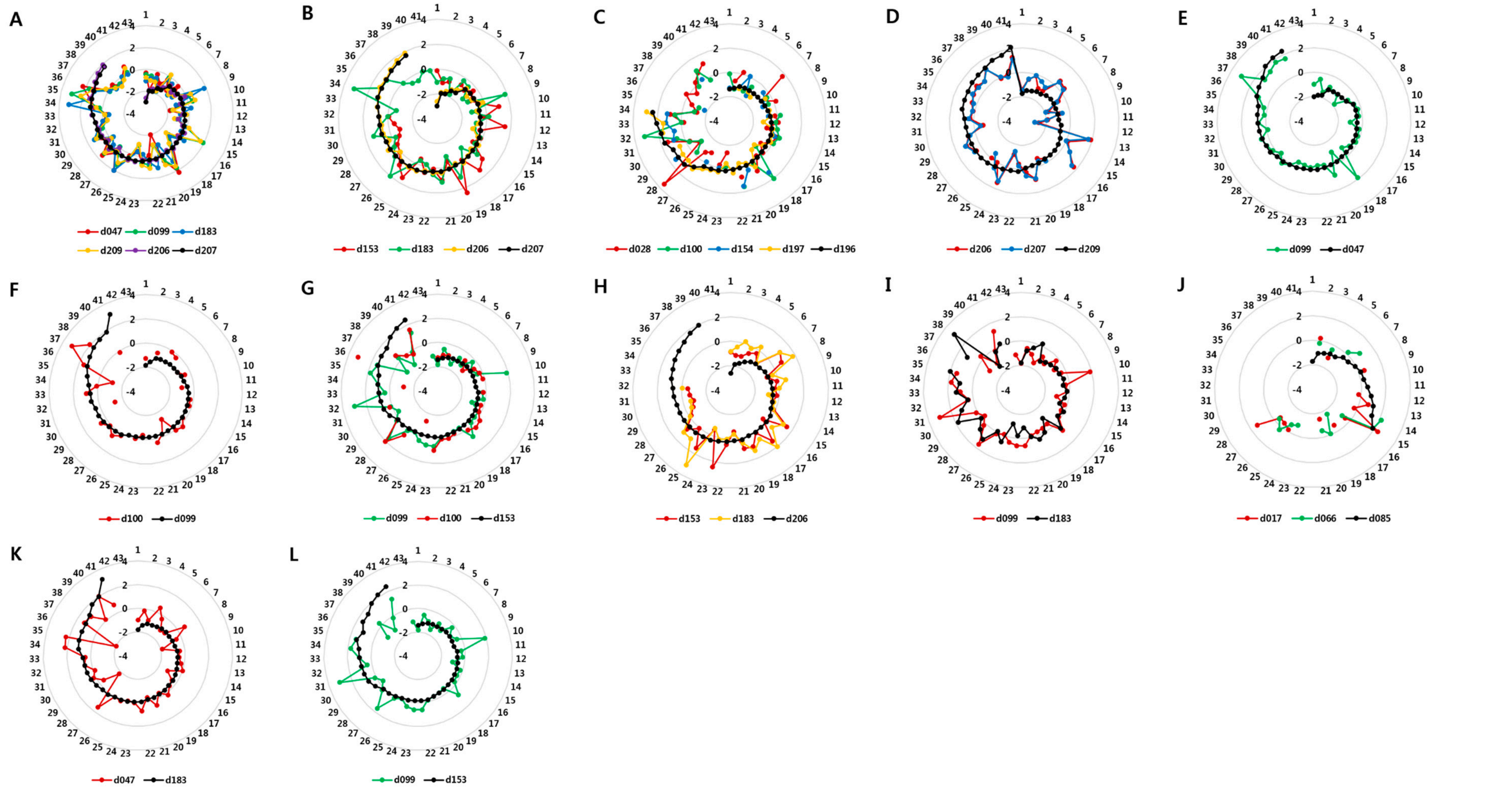

Figure S2. Radar charts of PBDE congeners detected in house dust by homologous group of PBDE formulations <extended>. The values from the center indicate log transformed PBDE levels, standardized with a mean of zero and standard deviation of unity, and those around the circumference are observation numbers in ascending order of reference BDE (black dots and lines) levels, where the congener number is provided in the legend after the "d" character. For example "d047" corresponds to BDE 47 in dust.

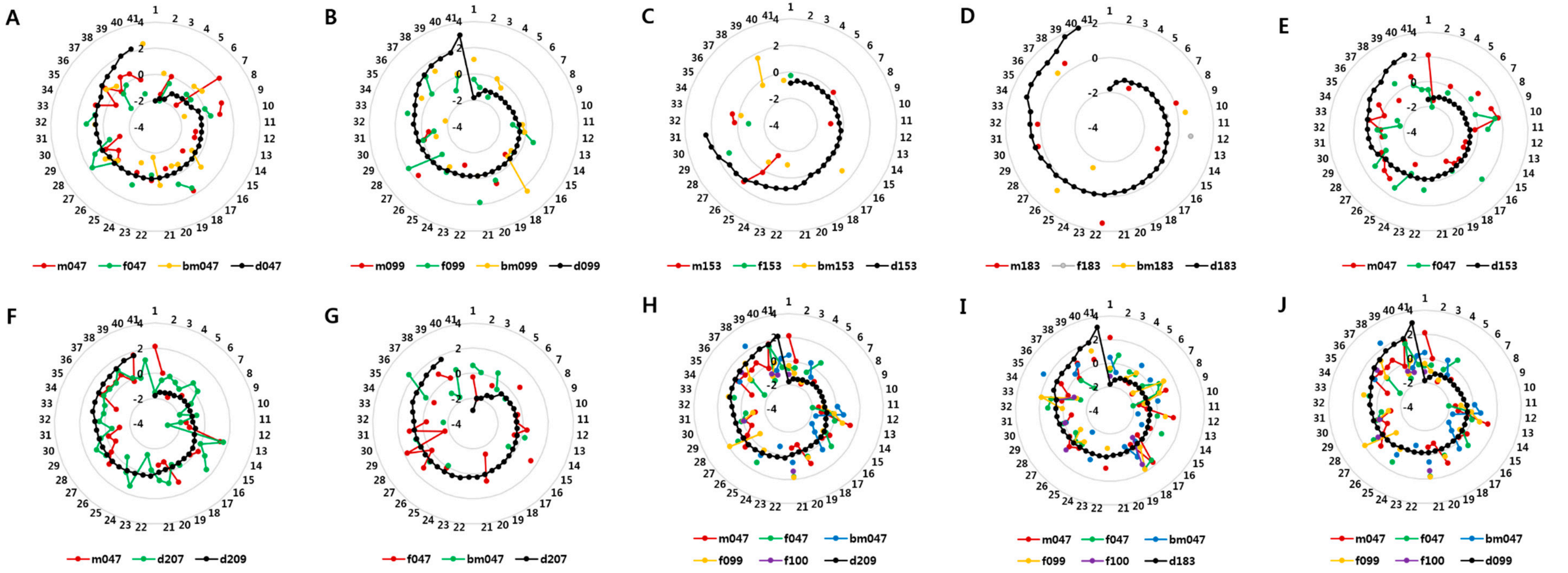

Figure S3. Radar charts of major PBDE congeners detected in house dust and major congener detected in human tissues <extended>. The values from the center indicate log transformed PBDE levels, standardized with a mean of zero and standard deviation of unity, and those around the circumference are observation numbers in ascending order of reference BDE (d209) levels, where the congener number is provided in the legend after the "m, f, bm and d" character. m; maternal serum, f; umbilical cord serum, bm; breast milk, d; house dust. For example "m047" corresponds to BDE 47 in maternal serum.

© 2016 by the authors; licensee MDPI, Basel, Switzerland. This article is an open access article distributed under the terms and conditions of the Creative Commons by Attribution (CC-BY) license (http://creativecommons.org/licenses/by/4.0/).
